# AI-powered prediction model for neoadjuvant chemotherapy efficacy: comprehensive analysis of breast cancer histological images

**DOI:** 10.1038/s41698-025-01033-1

**Published:** 2025-07-15

**Authors:** Fengling Li, Yani Wei, Wenchuan Zhang, Yuanyuan Zhao, Jing Fu, Xiuli Xiao, Yan Qiu, Yuhao Yi, Yongquan Yang, Hong Bu

**Affiliations:** 1 Department of Pathology, West China Hospital, Sichuan University, Chengdu, China; 2 Institute of Clinical Pathology, West China Hospital, Sichuan University, Chengdu, China; 3 Department of Pathology, The First Affiliated Hospital, Sun Yat-sen University, Guangzhou, China; 4 Department of Pathology, Shanxi Province Cancer Hospital/Shanxi Hospital Affiliated to Cancer Hospital, Chinese Academy of Medical Sciences/Cancer Hospital Affiliated to Shanxi Medical University, Taiyuan, China; 5 Department of Pathology, Sichuan Provincial People’s Hospital, Chengdu, China; 6 Department of Pathology, The Affiliated Hospital of Southwest Medical University, Luzhou, China

**Keywords:** Cancer, Predictive markers, Risk factors

## Abstract

Breast cancer patients exhibit variable responses to neoadjuvant therapy (NAT), necessitating robust predictive biomarkers. We developed an artificial intelligence (AI)-driven integrated predictive model (IPM) combining histopathological, clinical, and immune features to address this challenge. Using whole-slide images from 1035 patients across four centers, we compared tumor epithelium (TE-score), stroma (TS-score), and whole-tumor (TR-score) deep learning biomarkers, identifying TR-score as optimal (AUC = 0.729 vs. 0.686/0.719 for TE/TS-scores). The IPM, incorporating TR-score and clinical variables, demonstrated superior NAT response prediction versus clinicopathological models (validation AUC = 0.780 vs. 0.706, *p* < 0.001), with 10% higher accuracy. Immune profiling further enhanced performance (AUC = 0.831 vs. 0.822, *p* = 0.183). These results establish the biological and clinical validity of TR-score for characterizing tumor-stroma interactions, with IPM providing a generalizable framework for precision oncology. The model’s stability across multicenter cohorts (AUCs 0.781–0.816) and incremental value of immune data suggest its utility in guiding NAT decision-making and trial stratification.

## Introduction

Neoadjuvant therapy (NAT) is currently one of the important means for the clinical treatment of breast cancer. It can reduce tumor size and tumor burden, thus allowing inoperable patients to have access to surgical treatment^1^. Among breast cancer patients who receive NAT, those who achieve pathological complete response (pCR) after surgery may have more obvious survival benefits^2,3^. With the gradual standardization of clinical management, the pCR rate after NAT has improved, but some patients still have a poor response and even drug toxic reactions or disease progression^4,5^. Therefore, early prediction of NAT efficacy can guide clinical treatment decisions and is highly important for improving the prognosis of patients. Currently, clinical decision-making mainly relies on the molecular subtype and clinical stage of breast cancer. However, their ability to predict NAT is limited and cannot satisfy the demand of precise stratification. Therefore, the identification of novel and valuable prediction biomarkers has become an urgent problem in the field of neoadjuvant therapy for breast cancer.

Over the past few decades, most biomarkers in oncology have been discovered in the molecular biology field, whereas advances in computer technology have facilitated the direct extraction of hidden information from routinely available data^6^, such as medical images. Deep learning (DL) is a method in the field of artificial intelligence (AI) that uses artificial neural networks to identify repetitive patterns in complex datasets^7^. Since medical image data have high information density, the application of the DL algorithm to mine the underlying information of images can provide important value for clinical treatment. At present, AI-based image analysis has a wide range of applications in multiple medical fields that already involve image data, including radiomics and pathomics. In contrast to imaging methods, histology is a pervasive source that delivers high density of information from routine clinical practice. Breast cancer histomorphology is an important source of predictive information, and it has been reported that the underlying histological information extracted from pathological images via machine learning (ML) algorithms can assist in clinical diagnosis^8^, efficacy evaluation^9–11^ and prognosis prediction^12^. In the past several years, we have found that breast cancer histology contains rich predictive information, not only in the tumor epithelium (TE) epithelium^13^ but also in the tumor-associated stroma (TS)^14^, which suggests that DL can achieve in-depth mining of WSI information and comprehensive analysis of breast cancer tumor regions (including TE and TS). By taking into account the description of histological morphology characteristics, the model provides more abundant pathomic information for the prediction of NAT efficacy.

In routine pathological work, pathologists make diagnoses that rely not only on pathological data but also on clinical information, which indicates the importance of integrating multiple types of information. Breast cancer is a complex ecosystem composed of malignant cells and the tumor microenvironment, and its overall composition and related information are conducive to predicting NAT efficacy^15^. Liu et al. combined multimodal MRI features with clinicopathological risk factors to predict breast cancer pCR^16^, which outperformed the use of a single clinical factor. Sammut et al. improved the efficacy of NAT efficacy prediction for breast cancer by integrating multiomics information^15^. The above findings suggest that the information provided by a single layer is relatively limited and that the development of a multimodal prediction model combining tumor-related data of different dimensions may provide more reliable information for predicting the NAT response of patients with breast cancer. During the entire process of clinical management of breast cancer patients, pathological slides and routine clinical and examination data can be obtained from almost everyone. On the basis of the above easily accessible and informative data sources, a multimodal prediction model was constructed, which has great clinical application prospects in the field of NAT for breast cancer. At present, there are few reports on NAT prediction in patients with breast cancer.

**Fig. 1:**
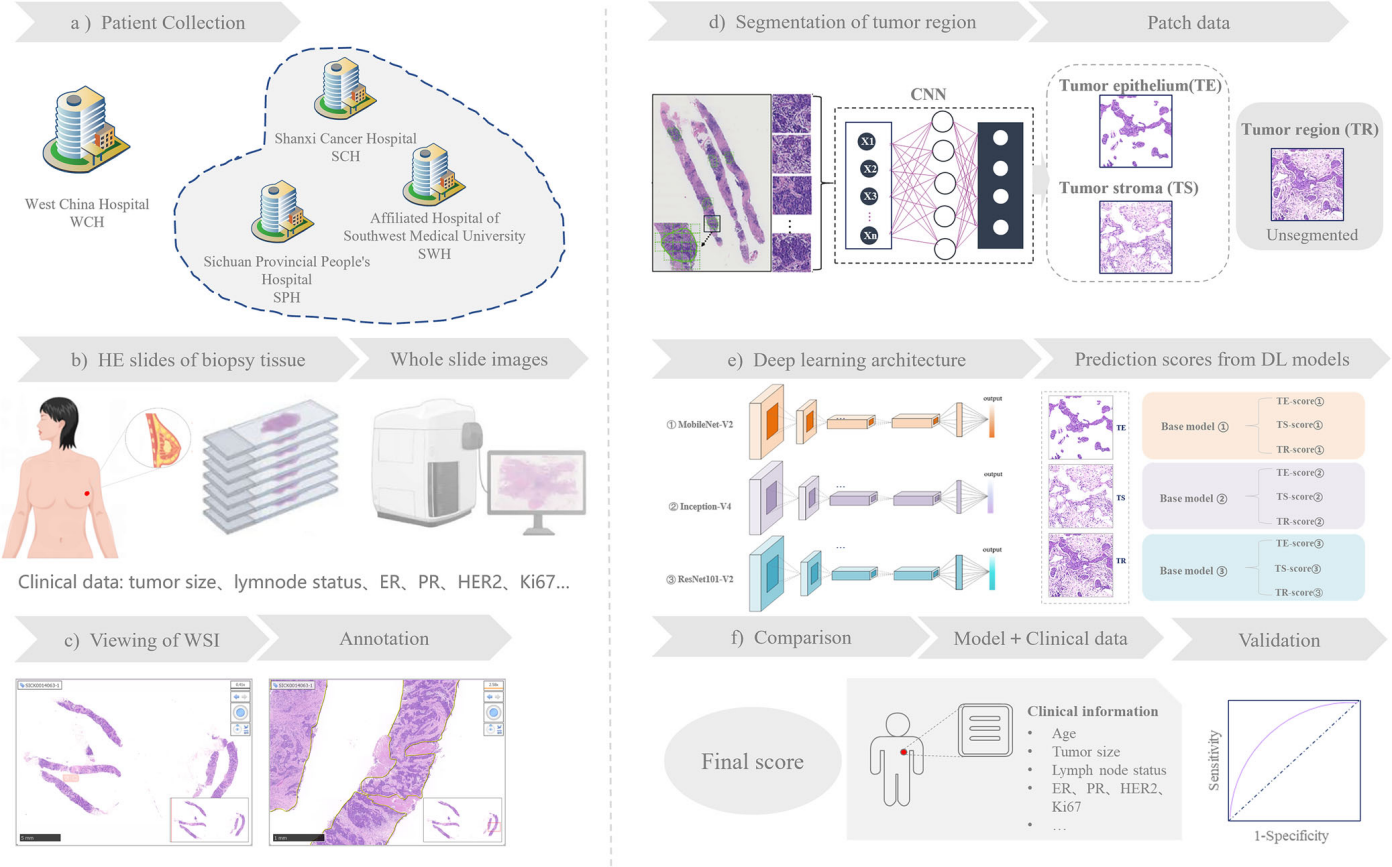
Study design for developing an AI-powered prediction model for pCR. **a** An overview of the datasets used: we included patients from four independent hospitals, cases from West China Hospital were used for prediction model fitting, and the other three datasets were independent validation cohorts. Pre-diagnostic risk factor exposure data were available for all datasets. **b**–**e** Our workflow for image processing: HE-stained slides of breast cancer biopsies are digitally scanned into WSIs, which are manually annotated, and the ROIs are cropped as patches (256 × 256 pixels at ×10 magnification); the CNN architecture is employed to segment the epithelial and stromal regions of the patches, leading to three types of patch data: pure TE patches, pure TS patches, and unified tumor area patches; three DL-based algorithms perform pCR-related histological information extraction on the basis of TE, TS, and TR, yielding nine different predictive scores. **f** The ML algorithm is used to integrate the selected histological factors with clinicopathological predictors to establish a prediction model. ER estrogen receptor, PR progesterone receptor, HER2 human Epidermal Growth Factor Receptor 2, WSI whole slide image, CNN convolutional neural network.

In fact, the immune system of patients with breast cancer is closely related to the prognosis and curative effect prediction of patients with breast cancer. Studies have reported the important role of stromal tumor-infiltrating lymphocytes (sTILs) in breast cancer^17–19^. However, little is known about non-sTIL immune cells. Therefore, this study intends to collect multicenter breast cancer cases and apply the DL algorithm to explore the predictive value of underlying tissue morphological information to perform a comprehensive analysis of the WSI. Histological markers generated by DL were integrated with clinicopathological information to construct an efficient pCR model for predicting breast cancer, improving the risk stratification of breast cancer patients. In addition, we incorporate immune cell infiltration information to enhance the prediction ability of the DL model that forms the basis of the integrated prediction model (IPM) (Fig. 1).

## Results

### Selection of the candidate histological score

**Fig. 2:**
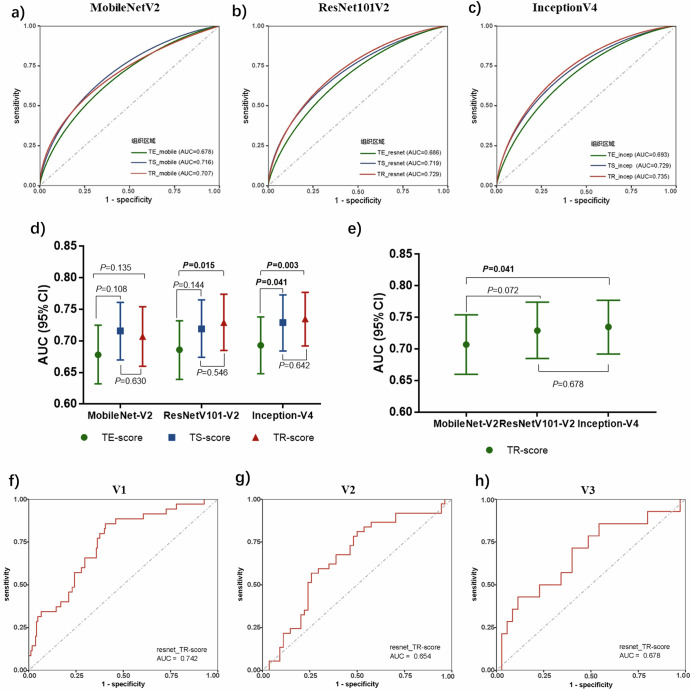
Comparison of the prediction ability of nine DL-based scores for DC. **a**–**c** ROC plots for predicting pCR from different tissue regions using MobileNet-V2, Inception-V4, and ResNet101-V2 as the algorithm basis. **d** Comparison of AUC values of predictive scores generated by different tissue regions (TE, TS and TR) in the same DL algorithm group: the predictive performance of the TE-score was poor. **e** Comparison of AUC values of TR-scores generated in three different DL algorithms for predicting pCR. **f**–**h** The selected TR-score based on ResNet101-V2 in the validation dataset for predicting pCR. TE tumor epithelium, TS tumor-associated stroma, TR tumor region.

A total of 1035 patients from four hospitals were included in our study (see Supplementary Table 5). Comparisons of the TE-score, TS-score, and TR-score were performed for DC. Compared with the TR-score and TS-score, the TE-score presented poorer predictive ability for all three algorithms (AUC = 0.678, 0.686, 0.693) (Fig. 2a–c). In the comparison of the TS-score and TR-score, the former showed a slight advantage over MobileNetV2 (0.716 vs. 0.707), whereas for the other two algorithms, the AUC values of the TS-score were lower than those of the TR-score (0.719 vs. 0.729; 0.729 vs. 0.735) (Fig. 2d). We subsequently compared the TR-scores of three different algorithms. The MobileNet-V2 model (AUC = 0.707) underperformed among the three, whereas no significant difference in performance was observed for ResNet101-V2 (AUC = 0.729) and Inception-V4 (AUC = 0.735) (Fig. 2e). Considering the performance and algorithm complexity, the TR-score based on ResNet101-V2 was selected as the candidate from the nine scores. Figure 2f–h shows the ROC curves of the TR-score for predicting pCR at V1-V3. Furthermore, the stratified analyses of TR-score’s predictive performance for different molecular subtypes of breast cancer were showed in Supplementary Fig. 3.

### Pathological observation of DL-based histological score

**Fig. 3:**
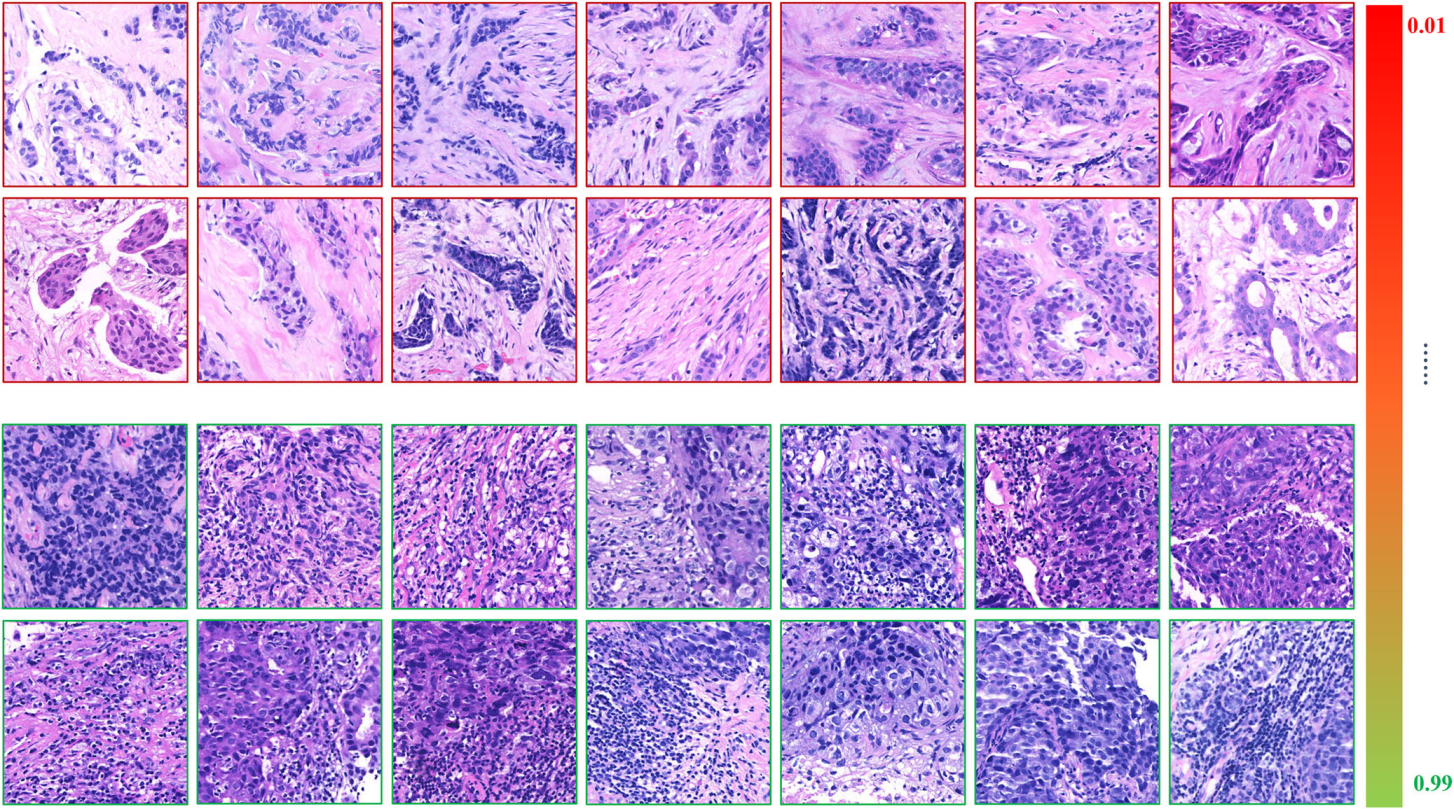
Histological characteristics varied between patches of high and low TR-scores. Low TR-score patches (upper) showed more uniform nuclei, less nuclear atypia, and tumor epithelial cells arranged in nests or cord-like structures, accompanied by abundant fibroblasts and collagen fibers in the stroma. High TR-score patches (bottom) exhibited darker tumor nuclei staining, greater nuclear atypia, frequent mitotic figures, and prominent perinuclear halos. The tumor epithelium grew in solid sheets with abundant lymphocytic infiltration in both epithelial and stromal regions.

For revealing the “black box” and insights into models, we performed an interpretability analysis to explore the morphological differences reflected by high and low TR-scores. We extracted patches corresponding to extremely high and extremely low TR-scores for histopathological analysis, aiming to understand the decision-making basis of the DL algorithm when generating TR-scores from tumor regions (Fig. 3). Comparative analysis revealed distinct morphological differences between the two groups: (1) high TR-score patches exhibited darker tumor nuclei staining, greater nuclear atypia, frequent mitotic figures, and prominent perinuclear halos. The tumor epithelium grew in solid sheets with abundant lymphocytic infiltration in both epithelial and stromal regions; (2) low TR-score patches, in contrast, showed more uniform nuclei, less nuclear atypia, and tumor epithelial cells arranged in nests or cord-like structures, accompanied by abundant fibroblasts and collagen fibers in the stroma. These findings align with our previous research^14,20^.

### Model construction and IPM prediction performance evaluation

**Fig. 4:**
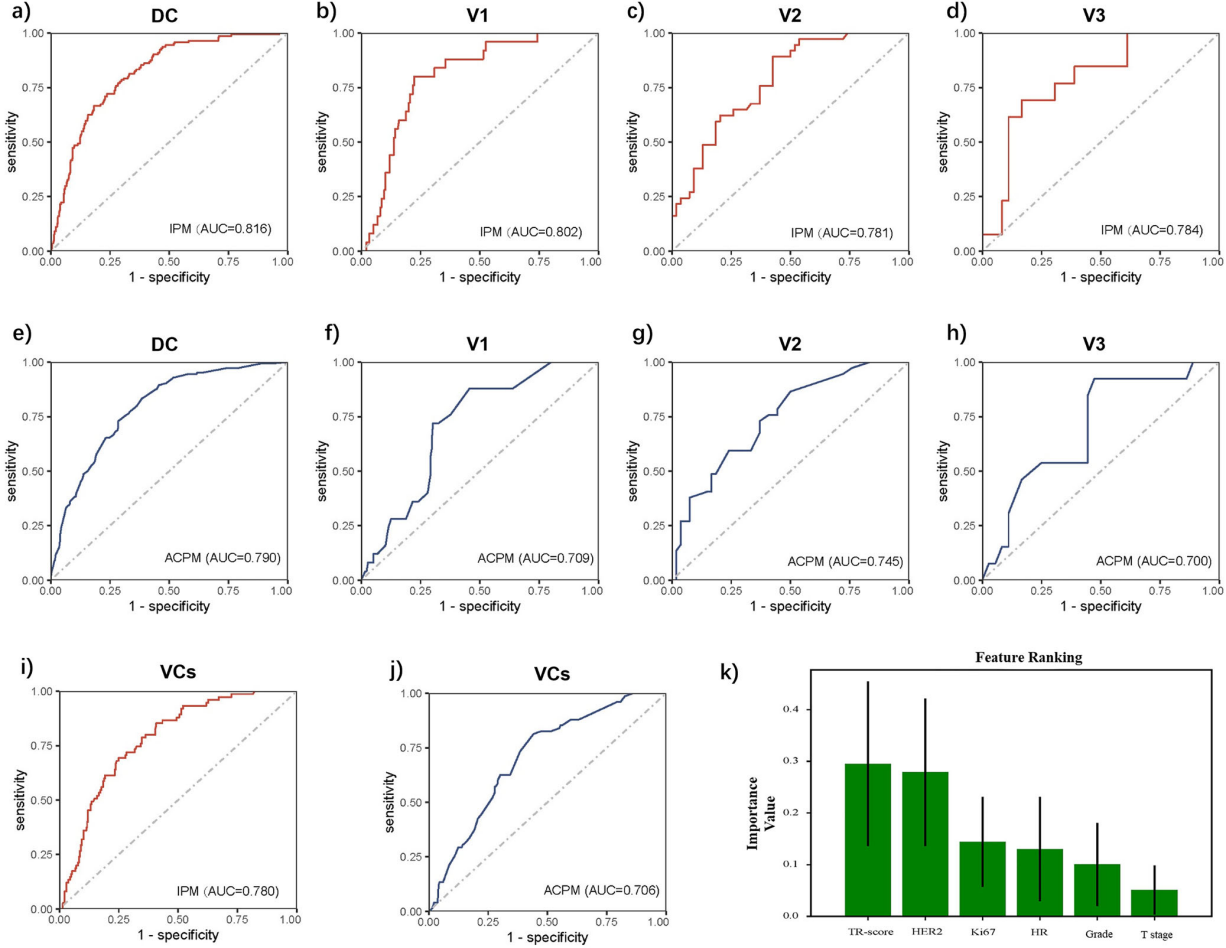
The prediction performance of the IPM and ACPM methods. **a**–**d** ROC plot of the IPM for predicting pCR in four datasets; **e**–**h** The clinical prediction model ACPM for the four datasets centrally predicted the pCR ROC diagram; the ACPM model yielded AUC values higher than 0.7 in the four datasets but lower than those of the IPM. Especially for the V1 data, the IPM AUC was as high as 0.802, which was significantly greater than that of ACPM (*p* < 0.001). **i**, **j** Show the performance of IPM and ACPM in the overall validation set, respectively, and the AUC of IPM was significantly greater than that of ACPM (*p* < 0.001). **k** Shows the feature ranking of IPM. DC discovery cohort, VCs validation cohort, TR tumor region, HER2 human Epidermal Growth Factor Receptor 2, HR hormone receptor.

Univariate logistic regression analysis revealed T-staging (*p* = 0.002), HER2 status (*p* < 0.001), HR status (*p* < 0.001), grading (*p* < 0.001), Ki67 (*p* = 0.005), and TR-score (*p* < 0.001) were significantly correlated with pCR (Supplementary Table 6). Therefore, the above six factors were integrated to construct an integrated prediction model, namely, IPM (the parameters of the random forest for combining the DL score and clinical factors were available in Supplementary Table 7). The top three factors in the importance ranking of the model features were the TR-score, HER2 expression and Ki67 expression, among which the TR-score ranked first among all the predictors (Fig. 4k). The IPM in DC reached an AUC of 0.816 (95% CI, 0.787–0.861) for predicting pCR in DC; in the external dataset of V1, the AUC was 0.802 (95% CI 0.755–0.849); and in the other two validation datasets, the AUC values were all greater than 0.780 (V2: 0.781, 95% CI 0.751–0.826; V3: 0.784, 95% CI 0.745–0.822) (Fig. 4a–d). According to the prediction of IPM, breast cancer patients could be classified into sensitive (Sen) and nonsensitive (Nonsen) groups; in the DC and VC groups, the pCR rates of the sensitive group were higher than 40%, which was significantly higher than those of the nonsensitive group (41.0% vs 7.8%, *p* < 0.001; 41.3% vs. 10.7%, *p* < 0.001) (Supplementary Note I and Fig. 4a, b). Confusion matrix of IPM for predicting pCR in three external validation sets were available in Supplementary Fig. 5.

Neo! adjuvant^21^ was reported to predict the probability of pCR in breast cancer patients receiving preoperative chemotherapy. Following a similar approach, we developed an alternative clinical prediction model (ACPM) using logistic regression and stepwise methods. The ACPM model included ER status, grade, T stage, and the NAT regimen as clinical factors. As shown in Fig. 4e–h, the ACPM algorithm yielded AUC values higher than 0.7 for the four datasets, but all the values were lower than those of the IPM. Especially in V1, the AUC of the IPM was 0.802, which was significantly greater than that of the ACPM algorithm (0.709, *p* < 0.001). However, for DC, V2 and V3, the difference in the AUC between the IPM and ACPM methods was not statistically significant (*p* = 0.094, *p* = 0.256, *p* = 0.300). In the validation set (VC), the AUC of the IPM for predicting pCR was 0.780 (95% CI 0.726–0.833), which was significantly greater than that of the ACPM (AUC = 0.706, 95% CI 0.645–0.768) (*p* < 0.001) (Fig. 4i, j). Additionally, a comparison of the classification performance between IPM and ACPM is shown in Supplementary Note II. The decision curves (Supplementary Fig. 6) demonstrate that IPM consistently shows superior net benefit across a wide range of clinical decision thresholds compared to ACPM, observed in both the discovery and validation settings. This finding suggests that IPM may possess greater clinical utility than ACPM for predicting neoadjuvant therapy response in breast cancer patients.

### Analysis of the prediction results

**Fig. 5:**
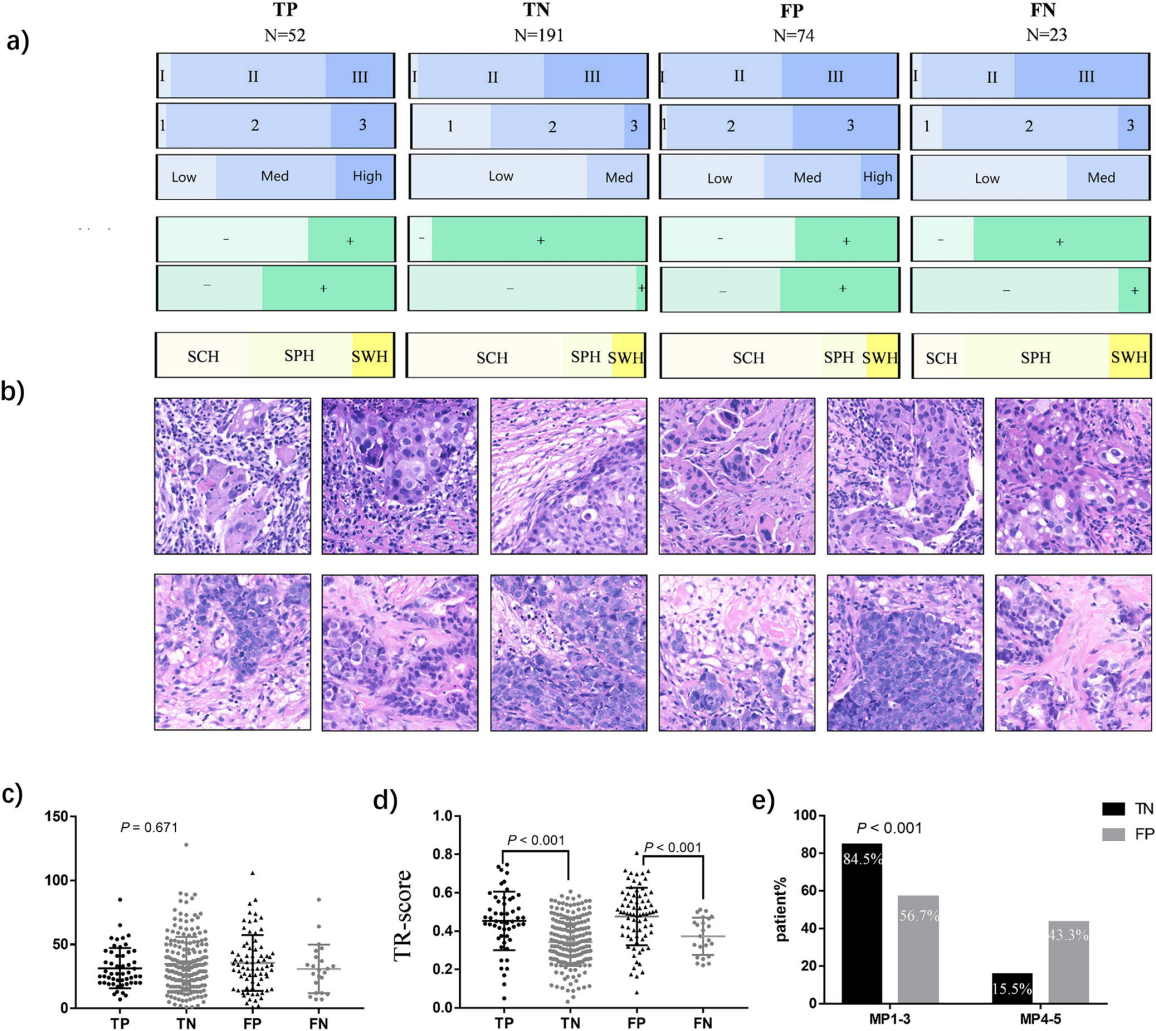
Analysis of IPM classification results. **a** The distribution of the four groups (TP/FP/TN/FN) of cases predicted by IPM; **b** Representative tiles of cases in the incorrect prediction group (FN and FP); **c** Distribution of the number of Tiles in the four groups; **d** Distribution of TR-scores among the four groups; **e** Comparative distribution of Miller-Payne grades in true non-responders (TN) vs. false positives (FP), demonstrating significantly greater MP4-5 frequency in FP cases (43.3% vs. 15.5%, *p* < 0.001), supporting IPM’s biological plausibility. TP true positive, TN true negative, FP false positive, FN false negative, MP miller-Payne.

The distributions of true positive (TP), false positive (FP), true negative (TN), and false negative (FN) cases predicted by IPM across different clinical stages, histological grades, sTILs, HR statuses, HER2 statuses, and institutions are shown in Fig. 5. Notably, the distribution of misclassified cases (FPs and FNs) differed among the three external institutions (*p* < 0.001). In the FP group, the proportion of SCH was the largest (SCH: 67.6%, SPH: 18.9%, SWH: 13.5%), whereas in the FN group, the proportion of SPH was the largest (SCH: 21.7%, SPH: 60.9%, SWH: 17.4%) (Fig. 5a). This finding indicates that most FP cases are from SCH, whereas more FN cases are from SPH. Patches from FP patients presented greater lymphocyte infiltration, higher grades, greater atypia, and high TE-score (Fig. 5b); in contrast, those from FN patients presented milder tumor cells and more collagen in the stroma, which was in accordance with our previous findings^13,14^. As shown in Fig. 5d, the distribution of TR-score in the FP group was significantly greater than that in the FN group (*p* < 0.001) but closer to that in the TP group (*p* < 0.001). Finally, we compared the number of tiles used in the four groups and found that there was no statistically significant difference in the number of tiles between the correct and incorrect prediction groups (*p* = 0.671) (Fig. 5c).

On the basis of the above analysis, the characteristics of sTILs, HR, grade, HER2 status and TR-score all provided power for IPM prediction, whereas the IPM prediction error was independent of the number of patches. We then analyzed whether there was any difference in the MP grading of patients in the TN and FP groups (Fig. 5e and Supplementary Table 5). The MP grades of all the patients were not available because of incomplete data. There were 142 patients (142/191) with MP grades in the TN group and 60 patients (60/74) in the FP group. We found that among the non-pCR samples, only 15.5% in the TN group had MP 4–5 cases, which was significantly lower than the 43.3% reported in the FP group (*p* < 0.001). This finding indicates that the cases were predicted to be NAT sensitive by IPM but actually non-pCR; although they did not achieve pCR in the end, they also had a relatively obvious pathological response to NAT. Otherwise, the difference of histological patterns between patches of high and low TR-scores was present in the Supplementary Fig. 6.

### The improvement of immune information

**Fig. 6:**
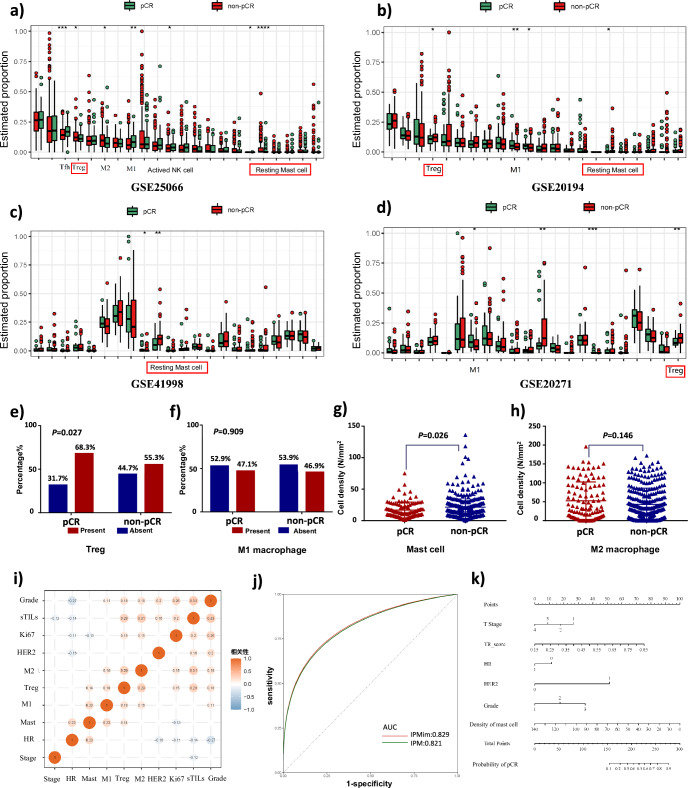
The improvement of immune information. **a**–**d** According to the CIBERSORT results, the distributions of each immune cell type in the pCR group and the non-pCR group were compared via the Mann‒Whitney test in the four GEO datasets. Three types of immune cells were included in the study, namely, Tregs, mast cells, and M1 macrophages. Each cell subtype was differentially distributed between the pCR and non-pCR groups in three or more datasets. **e**–**h** The four immune cell types were tested by IHC staining, and their distributions in the pCR and non-pCR groups were compared with the Mann‒Whitney or Pearson tests *χ*^2^. **i** The correlations between immune cells and clinical information are shown. **j** ROC curves of IPMim and IPM. **k** Nomogram of IPMim. M1 M1 macrophages, M2 M2 macrophages, Tregs regulatory T cells, pCR pathological complete response, HER2 human Epidermal Growth Factor Receptor 2, HR hormone receptor, sTILs stromal tumor-infiltrating lymphocytes, M1 M1 macrophages, M2 M2 macrophages; * represents *p* < 0.05, ** represents *p* < 0.01, and *** represents *p* < 0.001.

After screening from public databases, the following four datasets from the GEO database were included in this experiment: GSE25066 (*N* = 488), GSE20194 (*N* = 278), GSE20271 (*N* = 164) and GSE41998 (*N* = 121) (Supplementary Table 3). According to the CIBERSORT results (Fig. 6 and Supplementary Note III) and previous findings^22–25^, four types of immune cells were selected for the following experiments: regulatory T cells (Tregs), resting mast cells, M1 macrophages, and M2 macrophages (Fig. 6a–d), which are likely to be predictive of pCR. The four immune cells were tested by IHC staining in 388 breast cancer patients whose FFPE samples were available. By comparing their distributions in the pCR and non-pCR groups, we found that Treg and mast cells were significantly correlated with pCR (*p* = 0.027, *p* = 0.026), but M1 and M2 macrophages were not significantly correlated with pCR in this experiment (*p* = 0.909, *p* = 0.146) (Fig. 6e–h).

Through logistic regression and stepwise regression methods, we established an IPMim model that integrated immune information, the TR-score and clinicopathological information. The AUC of IPMim in predicting pCR was 0.831, while the original IPM without immune infiltration information was 0.822 (Fig. 6j). Delong test found that the AUC difference between the two did not reach statistical difference (*p* = 0.183). Compared with IPM, the NRI value was 0.025 (95% CI 0.031–0.080, *p* = 0.378), that is, IPMim improved the classification of pCR by 0.025. However, the IDI for comprehensive discrimination was improved by 0.017 (95% CI 0.003–0.030, *p* = 0.014). The nomogram was drawn according to the importance of the constituent factors of IPMim (Fig. 6k). It can be seen that only the density of mast cells in the immune infiltration cells was retained in the model, and its density was negatively correlated with the predicted probability of pCR.

## Discussion

In previous studies, we demonstrated the potential of both the tumor epithelium and the stroma for predicting the NAT response in patients with breast cancer; however, comprehensive image analysis and direct comparisons of TE and TS were not conducted. In the present study, we compared the predictive ability of histological scores derived from the TE (TE-score), TS (TS-score), and tumor region (TR-score) of WSIs with three DL algorithms. The TR-score, which considers TE and TS as a whole, exhibits superior performance as a candidate marker for modeling. Meanwhile, the predictive role of the TE region is relatively inferior to that of the TS and TR regions, suggesting that, compared with epithelial histological features, the stroma may provide more information for predicting therapeutic efficacy. This finding is similar to the findings of Beck et al. in their study on breast cancer prognosis, where quantitative information derived from the stroma was found to have a stronger correlation with survival rates than epithelial morphological characteristics^26^. In conventional pathological diagnosis, the diagnosis and grading of disease mainly rely on the morphological changes of epithelial components. In fact, the stroma plays an important role in tumorigenesis, development and metastasis. Bejnordi et al. found that stromal features could indicate the potential risk of ductal carcinoma in situ (DCIS) progressing to invasive ductal carcinoma (IDC)^27^, and our recent study reported that stromal collagen features correlated with NAC efficacy^28^. Hence, the tumor-associated stroma represents an understudied yet informative source of tumor-related information, and the application of computational approaches to stromal histology analysis may provide complementary predictive clues for diagnosis and response prediction. In the present study, according to the comparison of patches of high TR-score and low TR-scores (Fig. 3), both tumor epithelium and stroma contributed to the prediction ability of TR-score, but the most prominent feature we observed is TILs in stroma and epithelium. Therefore, TILs might be one of the reasons for that TR-score is superior to TE-score and TS-score, which is also supported by our previous studies^14,20^. Furthermore, isolated analysis of either epithelium or stroma can only provide limited tumor information. However, when analyzed as an integrated system, this approach enables simultaneous observation of both compartments while capturing their spatial interactions. Consequently, such comprehensive evaluation offers enhanced predictive value for treatment response prediction. Notably, compared with the TE-score generated in this experiment, the AUC for predicting pCR based on the epithelial score generated by the DL algorithm in preliminary experiments (Method 2.1) appeared to be greater (AUC = 0.847) (Result 3.2)^13^. There are two possible reasons for this discrepancy. First, the datasets are different: the former was validated in an internal validation set, whereas the TE-score in this experiment was validated in an independent external validation set. Second, the methodological strategies differ: the former generates a predictive score by identifying the TE-Tile with the highest probability across the entire WSI, whereas the latter is based on the TE region within manually selected ROIs. These findings suggest that both the selection of tissue regions and the number of samples impact the efficacy of the model/marker.

The proposed IPM integrates multiple clinical, pathological and DL image factors. Among the included metrics, the TR-score generated from histopathological images contributed the most to model prediction, indicating that the relevant quantitative representation of efficacy prediction extracted by the DL method has strong predictive potential. The IPM has been rigorously validated across three independent external datasets, outperforming the literature-based ACPM model^21^ in terms of comprehensive predictive and generalization capabilities. This enhanced performance is attributed to the integration of multiparameter predictive information to IPM, which provides a more comprehensive and quantitative characterization of tumor phenotypes compared with ACPM, which incorporates only a limited set of clinical and pathological factors. Additionally, the application of DL algorithms to digital pathology images offers robust predictive power, mitigating the impact of heterogeneous data on IPM performance. The IPM exhibits balanced performance metrics, with specificities greater than 0.7 in both the internal and external datasets, in contrast with the ACPM’s lower specificities of 0.615 and 0.558, which suggest a higher rate of false positives. Furthermore, the high NPV of IPM indicates that a substantial proportion of patients classified as nonresponders to NAT do not achieve pCR. This is particularly relevant in the context of breast cancer NAT, where all patients are at risk of chemotherapy-related toxicity, yet only a subset achieves pCR. Consequently, for patients identified as insensitive to NAT but susceptible to chemotherapy side effects, alternative treatment strategies may be considered, underscoring the need for a predictive model with a high NPV. In our study, all patients received NAT, with 551 out of 695 patients in the DC group not achieving pCR, and 265 out of 340 patients in the VCs group not achieving pCR. To analyze the actual value of IPM in clinical practice, we drew a decision curve (Supplementary Fig. 7), which shows that the net benefit of clinical decisions based on our model is of practical value compared to the “Treat-All” approach. Taking V1 as an example, the predicted classification results are as follows (pCR = 25, non-pCR = 175, confusion matrix: TP = 20, TN = 125, FP = 50, FN = 5). The current “treat-all” strategy has a net clinical benefit rate of −31.25%, reflecting that the majority of patients have undertreatment and should receive intensified treatment or other treatment options. The IPM model, through precise stratification, reverses the net benefit to +56.25% (Δ = 87.5%), mainly by avoiding undertreatment in 62 out of 100 patients. This improvement has significant clinical and economic value.

Currently, multiple studies have demonstrated that integrating imaging data with clinicopathological and genomic information can significantly enhance tumor classification and prognostic accuracy^16,29–32^. Building upon this evidence, we investigated whether incorporating immune infiltration features could further improve the predictive performance of our IPM model. The resulting IPMim, which included four immune cell subsets and sTILs as immune variables, showed moderately improved classification accuracy for pCR prediction compared to the original IPM, though this enhancement did not reach statistical significance. Notably, mast cell density emerged as the sole immune-derived component retained in the IPMim, a particularly intriguing finding given the limited existing reports on mast cells’ role in predicting neoadjuvant therapy response. While mast cells are established regulators of inflammatory processes^33^, their prognostic significance in cancer remains controversial, with studies reporting both favorable^34^ and adverse^35^ associations. This apparent contradiction may reflect context-dependent functional plasticity, as mast cell behavior appears influenced by breast cancer molecular subtypes and tumor grade^36,37^. Their functional diversity likely originates from their capacity to secrete various mediators in a microenvironment-dependent fashion^38^, enabling both pro-tumorigenic^39^ and tumor-suppressive effects^40^. Our finding of an inverse correlation between mast cell abundance and pCR aligns with Reddy et al.‘s observations in inflammatory breast cancer^41^, where spatial profiling suggested mast cells may promote treatment resistance through multiple mechanisms: suppression of CD8 + T cell activity, amplification of immunosuppressive CD163+ macrophages, and direct stimulation of tumor cell proliferation via both contact-dependent and paracrine signaling. Although further mechanistic validation is required, these collective findings position mast cells as a potential therapeutic target for enhancing NAT response, warranting deeper investigation into their multifaceted roles in breast cancer biology.

The multimodal model has become a trend in the field of artificial intelligence^42^. In a recently reported study^15^, Sammut et al. collected clinical, digital pathology, genomic, and transcriptomic features from pretreatment breast tumor biopsies of 168 patients and integrated these features into a multiomics machine learning model; the model achieved an AUC of up to 0.87 in predicting treatment effects in an external validation cohort (*n* = 75 patients). As emphasized in their study on the importance of multidimensional data integration for response prediction, a variety of information resources have given rise to a new direction of multimodal research^43^. In the clinical setting, data sources are abundant, such as basic patient information at admission, clinical diagnostic information, and pathological sections, all of which are relatively common and easily accessible data patterns. Compared with the model proposed by Sammut et al.^15^, the IPM proposed in this study uses clinical routine data sources that are readily available, thus having greater clinical practical value. Moreover, in the digital pathology information they included, only the lymphocytic infiltration information from the pathological images was extracted, while the TR-score in the IPM was derived from the comprehensive information extracted from the tumor area by DL algorithms. Furthermore, IPM was successfully validated across three independent external datasets. Therefore, the IPM has the potential to identify patients who are likely to benefit the most from NAT prior to treatment, thus sparing less responsive patients from unnecessary treatment and assisting in the development of more specific clinical NAT plans.

However, this study has several limitations. Although the IPM was independently validated in three external hospitals, it has not been prospectively evaluated. In addition to histological information and clinicopathological information, transcriptome features may also be predictive of NAT efficacy. However, the amount of biopsy tissue is small and precious, and the preservation quality of FFPE samples sometimes fail to meet sequencing requirements. Therefore, omics data are not available for relevant research.

Conclusively, we demonstrated that the tumor region, containing both the stroma and the epithelium, was superior to either the pure epithelium or the pure stroma in predicting treatment response and proposed an integrated model, the IPM, which is highly effective for the early prediction of breast cancer patients’ response to NAT. In addition, we found that the inclusion of immune infiltration information could improve the predictive power of the IPM, providing clues for further research on breast cancer pCR prediction.

## Methods

### Patient

Breast cancer patients receiving NAT from West China Hospital (WCH, 2010.01--2021.04), Shanxi Cancer Hospital (SCH, 2015.02--2019.10), Sichuan Provincial People’s Hospital (SPH, 2020.01--2021.02) and the Affiliated Hospital of Southwest Medical University (SWH, 2016.08--2020.10) were retrospectively enrolled (Fig. 1). Eligible subjects were screened according to established inclusion and exclusion criteria^14^. Clinicopathological information, such as tumor size, lymph node status, clinical stage, treatment regimen and immunohistochemistry test results, the NAT response, histological grade and sTILs were collected for the included patients. Hematoxylin‒eosin (HE)-stained sections and formalin-fixed and paraffin-embedded (FFPE) tissues were extracted from the breast biopsy tissue of the patient. Patients from WCH were assigned to the discovery cohort (DC), whereas other patients were assigned to three different validation cohorts (VC).

Our study was approved by the ethics committee of participating hospitals (No. 764 in 2021) and abided by the Declaration of Helsinki before using tissue samples for scientific research purposes only. The requirement to obtain informed consent from the participants was waived by the ethics committee.

### Image acquisition and algorithm processing

All the above HE sections were digitally scanned into whole slide images (WSIs) at ×40 magnification via a Hamamatsu scanner (NanoZoomer-XR C12002, Hamamatsu, Japan). The WSIs of each patient were manually annotated to the region of interest (ROI) by pathologists with an open tool (Aperio ImageScope), which refers to the representative tumor regions containing both TE and TS (Fig. 1). Annotations were performed by two board-certified pathologists (≥3 years breast pathology experience) with double verification. ROIs were cropped into patches (256 × 256 pixels) at 10× magnification, and the epithelium-stroma classifier (E‒S classifier) was employed to segment the TE and TS^14^. Three types of patch data were produced: pure TE patches, pure TS patches, and TR patches. Considering their variance in intrinsic characteristics, three DL algorithms (Inception-V4, MobileNet-V2, and ResNet101-V2) were selected as candidates for the basic architectures to perform WSI analysis for comparison (Supplementary Table 1), since we aimed to propose an effective and practical histological score based on a DL architecture that could reach a balance between complexity and inference speed (Fig. 1). Three established architectures: Inception-v4, MobileNet-v2, and ResNet101-v2 were employed as foundational deep learning backbones. The models were optimized using:

Loss function: weighted cross-entropy to address class imbalance^44^:

1 $$L=-[{w}_{1}\times t\times \log \left(p\right)+{w}_{0}\times \left(1-t\right)\times \log \left(1-p\right)]\,\,\,\,s.t.\,\,\,{w}_{0}+{w}_{1}=1$$

Stochastic Gradient Descent (SGD)^45^ was used for optimization and a rapid ensemble method was implemented to enhance CNN performance^46–48^. Finally, nine different histological scores were generated from the WSIs of TE, TS, and TR via Inception-V4, MobileNet-V2, and ResNet101-V2, respectively (the detailed methods are available in Li et al.^14^). Detailed documentation and source code deposited in the following repository: https://github.com/YongQuanYang/TS-Score.

### Building the integrated-prediction model

Nine histological scores from the experiments above were compared for their ability to predict pCR, and the most appropriate score was selected as the candidate biomarker for building the IPM. Univariate logistic regression analysis was used to screen the baseline clinicopathological parameters significantly related to pCR. The random forest algorithm from the scikit-learn package (version 1.0.2) was used to construct the IPM by combining the baseline predictors with DL-based histological scores. The best number of “n_estimators” was determined by drawing the learning curve, and other parameters, including cutoff, were determined by using the grid search method to achieve the highest area under the curve (AUC) in the training set. Tenfold cross-validation was performed on the DC data to search for the best parameter values, followed by independent validation on three external datasets. This part of the ML framework was built on Python (3.9.12). Our study adopted the following recommended Checklist for Artificial Intelligence in Medical Imaging (CLAIM) guidelines (Supplementary Note IV)^49^.

### CIBERSORT analysis

Download the four datasets from GEO website (https://www.ncbi.nlm.nih.gov/geo/) (Supplementary Table 3). CIBERSORT^50^ was used to calculate the abundance of 22 types of immune cells in each sample. CIBERSORT is a method to calculate the composition of immune cells in complex tissues based on gene expression, which has been shown to have a strong agreement with the true proportion in mixed tumor tissues^50,51^. We used a white blood cell genetic signature matrix called LM22, which contains 547 genes that distinguish 22 human hematopoietic cell phenotypes, including seven T cell types, naï B and memory B cells, plasma cells, natural killer (NK) cells, and myeloid subsets. CIBERSORT was combined with the LM22 eigenmatrix to predict the proportion of 22 immune cell types in each breast cancer sample in the four datasets. Based on CIBERSORT prediction results, the difference of immune cell proportion between pCR group and non-pCR group was compared in the four datasets.

### Immunohistochemistry validation

Mann–Whitney test was used to compare the different distribution of immune cells between the pCR group and the non-pCR group, and the intersection of different immune cell subsets in four independent datasets was analyzed. Immune cell subtypes with differential distribution between pCR and non-pCR groups in three or more datasets were considered as candidate subtypes for inclusion in the study. These include Tregs, mast cells, and M1 macrophages. In addition, considering the correlation between different subtypes of macrophages^22–25^ and the correlation between M2 macrophages and poor prognosis in breast cancer reported in the literature^25,52–54^, M2 macrophages from 22 immune cell types were included in this study for analysis. We performed IHC staining on the collected breast cancer FFPE tissue. First of all, the slice dewaxing, then antigen repair and closed, then add a resistant reagent and incubation for the night related immune cell phenotype in different antibody staining: Treg (Foxp3), mast cells (tryptase), M1 macrophages (iNOS), M2 macrophages (CD163). The optimal experimental conditions for IHC staining of the four indicators were obtained according to the results of the pre-experiment (Supplementary Table 4). After incubation plus two resistant reagent, then using DAB chromogenic reaction. Immunocytes were counted using a previously described and validated computer-aided counting method^55^ using the free image analysis software ImageJ^56^. The whole IHC stained sections were observed under a low magnification microscope, and more than three hot spots were selected under a 20x objective, and pictures were taken with a Sony microscope camera. We used ImageJ software to count positive cells based on their size, shape, and intensity of positivity. Treg/M1 macrophages were classified according to the presence or absence of positive cells: Treg/M1 macrophages were absent (Foxp/iNOS staining positive cells were absent), Treg/M1 macrophages were present (Foxp/iNOS staining positive cells were ≥1); The mean value of the counted fields represents mast-cell and M2 macrophage infiltration (Supplementary Fig. 1).

### Addition of immune information

In this part, we initially explored the role of immune information in improving the predictive efficiency of the IPM, aiming to provide a reference direction for the subsequent optimization of the model. Breast cancer sequencing datasets meeting the criteria were screened from public databases, the CIBERSORT^50^ algorithm was applied for batch analysis of gene expression profiles, and the proportions of 22 immune cell subsets were inferred. After screening PCR-related immune cell types, IHC technology was used to detect immune cells in FFPE samples to analyze the predictive value of the infiltration information of candidate immune cell subtypes for the NAT efficacy of breast cancer and to explore the improvement in the predictive ability of the IPM via the infiltration information of immune cells. Detailed information in this part is available in Supplementary Information II. To investigate whether tumor immune infiltration information could further improve the predictive performance of the IPM, we integrated the four immune cells detected by IHC and the manual assessment results of sTILs as a new dimension of information by logistic regression and stepwise regression with the deep histological information and baseline predictors proposed above, building a prediction model containing immune information (IPM with immune information, IPMim). The predictive ability of IPMim and IPM for pCR was subsequently compared to evaluate the improvement in model performance in terms of immune infiltration information.

### Statistical analysis

The ML algorithm was used to train and construct the model in DC, and the predictive capability of the model/score was evaluated via fivefold cross-validation in DC and independent validation in VC. The area under the curve (AUC), accuracy, specificity, sensitivity, positive predictive value (PPV), and negative predictive value (NPV) were used to evaluate the prediction ability of the model, and the Delong test was used to compare the AUC. The net reclassification improvement (NRI) and integrated discrimination improvement (IDI) were used to measure the improvement of the immune information to the model prediction effect.

## Supplementary information

Supplementary information

## Data Availability

The data used and/or analyzed during the current study are available from the corresponding author upon reasonable request.

## Code availability

The code required to reproduce these findings is available for download from https://github.com/YongQuanYang/TS-Score.
