# Supplementary material for: AI-powered prediction model for neoadjuvant chemotherapy efficacy: comprehensive analysis of breast cancer histological images
Source: NPJ Precis Oncol. 2025 Jul 15;9:239. doi: 10.1038/s41698-025-01033-1 (PMC12263982; doi:10.1038/s41698-025-01033-1)
Supplement: Supplementary file 1 — Supplementary information [file 41698_2025_1033_MOESM1_ESM.pdf]

Supplementary Table 1 Key information of the three deep learning algorithms

DL algorithm	Size (MB)	Parameter	Depth	Inference step time (CPU ms)	Inference step time (GPU ms)
MobileNet-V2	14	3.5M	105	25.9	3.8
ResNet101-V2	171	44.7M	205	72.7	5.4
Inception-V4	215	55.9M	449	130.2	10.0

* https://keras.io/api/applications/

Supplementary Table 2 The inclusion and exclusion criteria of GEO database

The inclusion criteria	The exclusion criteria
Primary invasive ductal breast cancers	Distant metastasis
Sequencing used primary breast cancer biopsy tissues	Not Affymetrix Gene chip
Standard NAT regimens	Lack of key clincal information
With pathological results of pCR or non-pCR	Samples < 120

Supplementary Table 3 Basic information of the four GEO datasets

GEO Dataset	Subtype	Sample size	Platform
GSE41998	All	121	GPL571
GSE25066	HER2-negative	488	GPL96
GSE20194	All	278	GPL96
GSE20271	All	164	GPL96

Supplementary Table 4 Experiment details of IHC staining

Antibody	Manufacturers	Clone number	Dilution ratio	Antigen repair pH
Tryptase	Abcam; Britain	ab2378	1:20000	9
Foxp3	Abcam; Britain	ab20034	1:500	6
CD163	Abcam; Britain	ab182422	1:400	9

Antibody	Manufacturers	Clone number	Dilution ratio	Antigen repair pH
iNOS	Abcam; Britain	ab283655	1:500	6

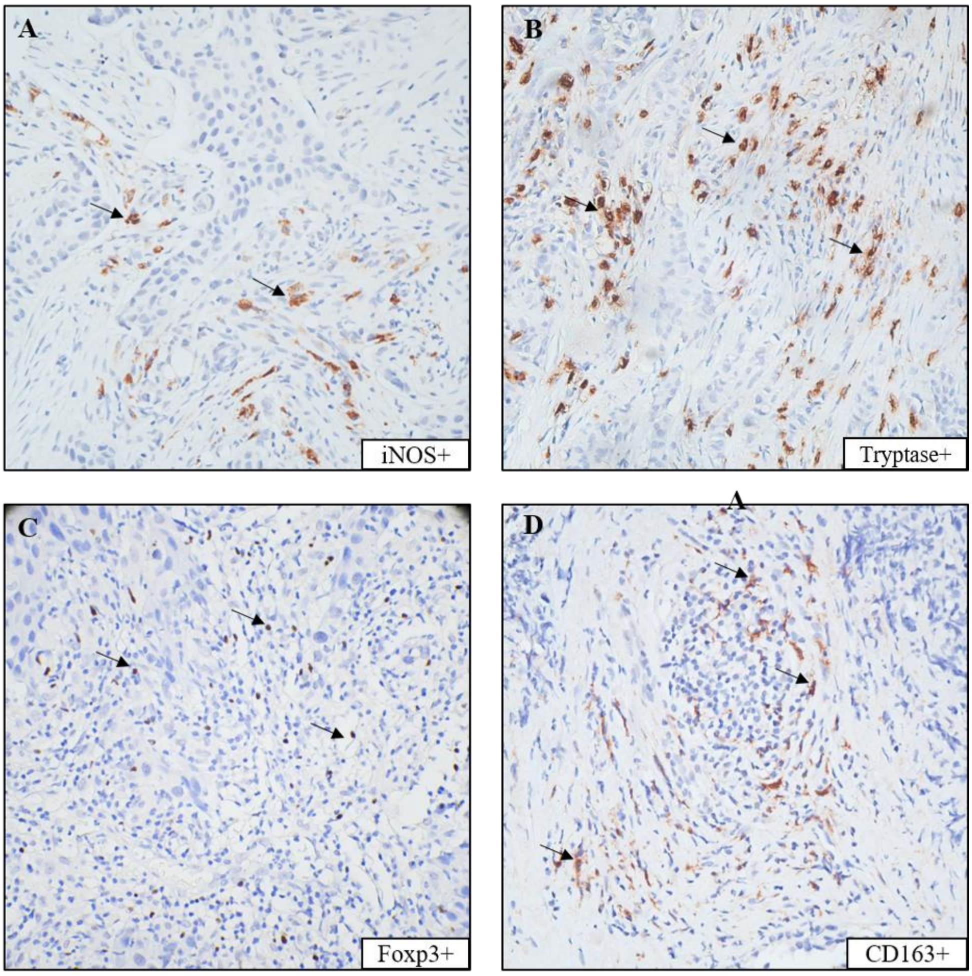

Supplementary Figure 1 IHC staining pictures

Supplementary Table 5 Clincopathological parameters of patients in four hospitals

**Parameters**		WCH N=695 (%)	SCH N=200 (%)	SPH N=91 (%)	SWH N=49 (%)
**Age**	*P-value**		**<0.001**	**0.002**	0.551
< 50		402 (57.8)	71 (35.5)	37 (40.7)	26 (53.1)
≥ 50		293 (42.2)	129 (64.5)	54 (59.3)	23 (46.9)
**T Stage**	*P-value**		**<0.001**	**0.01**	0.078
T1-T2		334 (48.1)	164 (82.0)	57 (62.6)	30 (61.2)

T3-T4		361 (51.9)	36 (18.0)	34 (37.4)	19 (38.8)
**N stage**			**<0.001**	**<0.001**	**<0.001**
N0		58 (8.3)	35 (17.5)	27 (29.7)	16 (32.7)
N1-N3		637 (91.7)	165 (82.5)	64 (70.3)	33 (67.3)
**HR**	*P-value*[*]		0.930	0.542	0.521
Negative		209 (30.1)	59 (41.8)	24 (26.4)	17 (53.1)
Positive		486 (69.9)	141 (58.2)	67 (73.6)	32 (46.9)
**HER2**	*P-value*[*]		**<0.001**	0.233	0.056
Negative		479 (68.9)	179 (89.5)	57 (62.6)	27 (55.1)
Positive		216 (31.1)	21 (10.5)	34 (37.4)	22 (44.9)
**Subtype**	*P-value*[*]		**<0.001**	0.481	**0.009**
HR+/HER2-		370 (53.2)	134 (67.0)	44 (48.4)	15 (30.6)
HER2+		216 (31.1)	20 (10.0)	34 (37.4)	22 (44.9)
TNBC		109 (15.7)	46 (23.0)	13 (14.2)	12 (24.5)
**Ki67 index**	*P-value*[*]		**0.001**	0.112	1.000
Low (< 20%)		100 (14.4)	12 (6.0)	18 (19.8)	7 (14.3)
High (≥ 20%)		595 (85.6)	188 (94.0)	68 (74.7)	42 (85.7)
Unknown		-	-	5 (5.5)	-
**Grade**	*P-value*[*]		**0.002**	**<0.001**	**0.022**
1/2		486 (69.9)	162 (81.0)	79 (86.8)	42 (85.7)
3		209 (30.1)	38 (19.0)	12 (13.2)	7 (14.3)
**sTILs**	*P-value*[*]		0.387	0.700	0.080
Low		397 (57.1)	125 (62.5)	53 (58.2)	20 (40.8)
Moderate		251 (36.1)	64 (32.0)	30 (33.0)	25 (51.0)
High		47 (6.8)	11 (5.5)	8 (8.8)	4 (8.2))
**pCR [1]**	*P-value*[*]		**0.045**	**0.001**	0.732
Yes		169 (24.3)	35 (17.5)	37 (40.7)	13 (26.5)
No		526 (75.7)	165 (82.5)	54 (59.3)	36 (73.5)
**pCR [2]**	*P-value*[*]		**0.008**	**<0.001**	0.365

Yes		144 (20.7)	25 (12.5)	37 (40.7)	13 (26.5)
No		551 (79.3)	175 (87.5)	54 (59.3)	36 (73.5)
**MP**	*P-value**		-	-	-
1-3		-	90 (45.0)	37 (40.7)	28 (57.1)
4/5		-	50 (25.0)	51 (56.0)	21 (42.9)
Unknown		-	60 (30.0)	3 (3.3)	0 (0.0)

P-value* refers the comparisons between WC and SCH/SPH/SWH

Supplementary Table 6 The univariate logistic regression analysis of the baseline factors correlated with pCR

		Discovery cohort		Univariate analysis		
		**pCR**<br>**N = 144**	**Non-pCR**<br>**N = 551**	**OR**	**95% CI**	***P***
**Age**	Median (range)	49(27-68)	48(24-76)	1.00	0.99-1.03	0.358
**T stage**				-	-	**0.002**
	T1	12(8.3)	24(4.4)	Ref	Ref	-
	T2	70(48.6)	228(41.4)	0.61	0.29-1.29	0.198
	T3	29(20.1)	83(15.1)	0.70	0.31-1.57	0.387
	T4	33(22.9)	216(39.2)	0.31	0.14-0.67	0.003
**N stage**				-	-	0.375
	N0	13(9.0)	45(8.2)	Ref	Ref	-
	N1	72(50.0)	235(42.6)	1.06	0.54-2.08	0.864
	N2	26(18.1)	121(22.0)	0.74	0.35-1.57	0.438
	N3	33(22.9)	150(27.2)	0.76	0.37-1.57	0.460
**HER2**	negative	51(35.4)	428(77.7)	6.35		**<0.001**

		Discovery cohort		Univariate analysis		
		**pCR N = 144**	**Non-pCR N = 551**	**OR**	**95% CI**	***P***
	positive	93(64.6)	123(22.3)		4.27-9.43	
**HR**	negative	77(53.5)	132(24.0)	0.27	0.19-0.40	**<0.001**
	positive	67(47.5)	406(76.0)			
**Ki67 index**	Median (range)	40(5-85)	40(2-95)	1.01	1.00-1.02	**0.005**
**grade**				-	-	**<0.001**
	low	10(6.9)	127(23.0)	Ref	Ref	-
	moderate	76(52.8)	326(59.2)	2.96	1.48-5.91	0.002
	high	58(40.3)	98(17.8)	7.52	3.66-15.5	<0.001
**TR-score**	Median (range)	0.47(0.14-0.81)	0.36(0.03-0.86)	121.0	33.6-435.5	**<0.001**

Supplementary Table 7 The parameters of the random forest for combining the DL score and clinical factors

Parameter	Setting
n_estimators	188
max_depth	4
min_samples_leaf	1
min_samples_split	2
max_features	2
class_weight	balanced

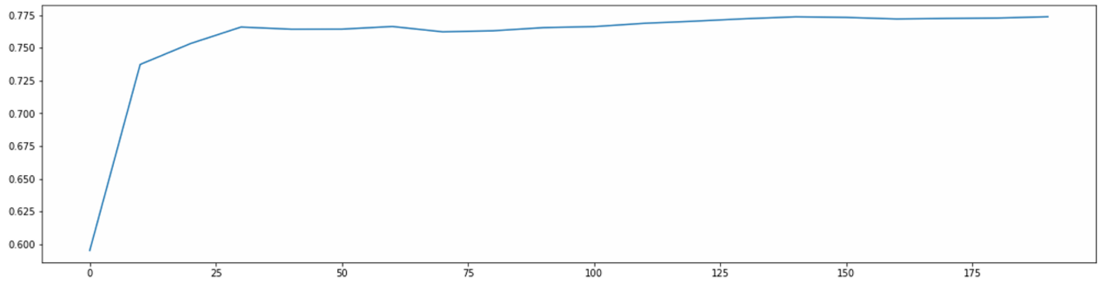

Supplementary Figure 2 Wrapper method-based learning curve demonstrating the relationship between the number of trees (n_estimators, x-axis) and model performance (5-fold cross-validation AUC, y-axis). Performance plateaus at approximately n_estimators=188, indicating optimal parameter selection.

Supplementary Note I

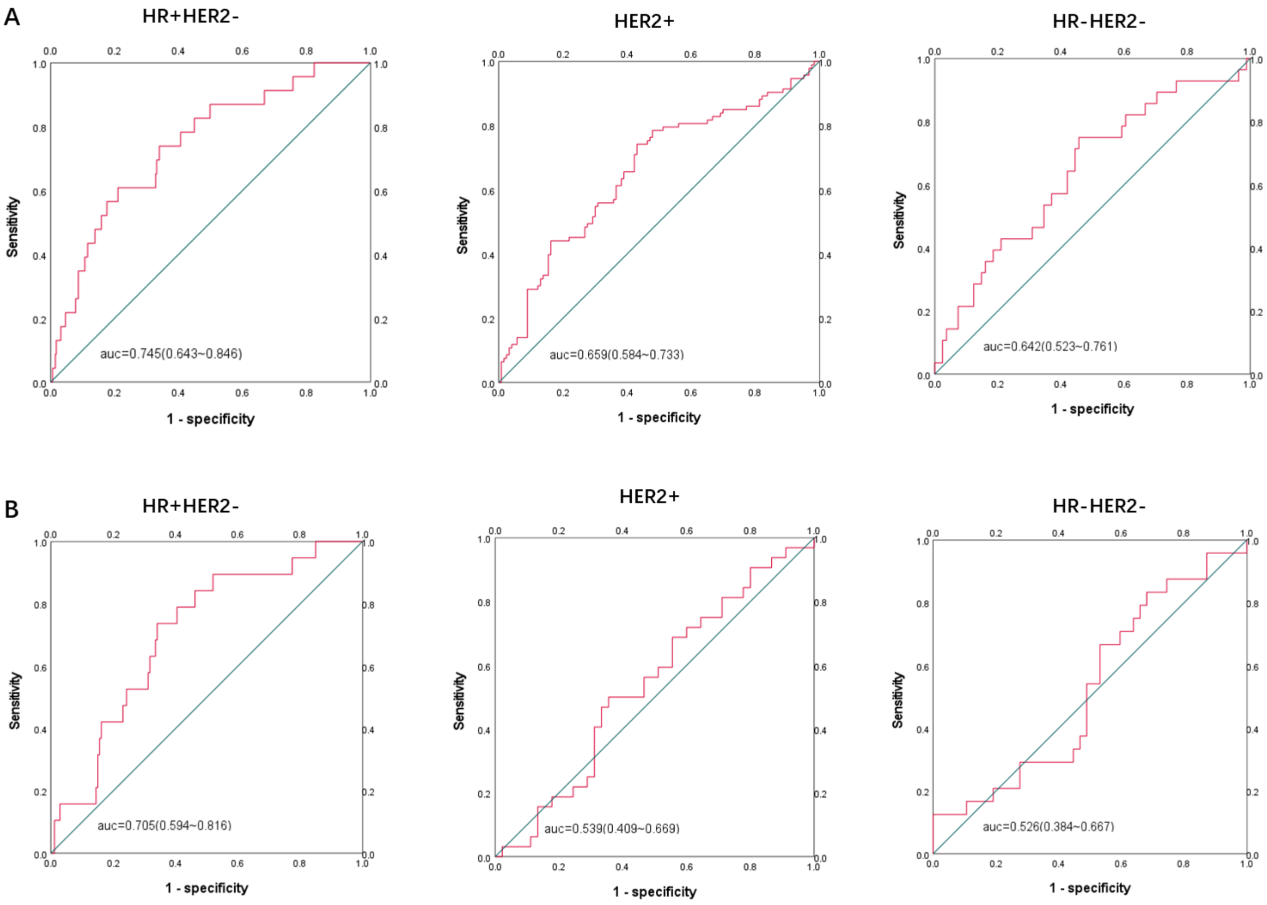

Supplementary Figure 3 The stratified analyses of TR-score different subtypes

ROC curves of TR-score among different breast cancer subtypes in WCH (A) and the extra three hospitals (B)

According to the prediction of IPM, breast cancer patients could be classified into sensitive (Sen)

group and non-sensitive (Non-sen) group; in DC and VCs, the pCR rates of the sensitive group were higher than 40%, which was significantly higher than those of the non-sensitive group (41.0% vs 7.8%, $P < 0.001$; 41.3% vs 10.7%, $P < 0.001$) (Supplementary Figure 4 A-B). Similarly, in both HR-positive and HR-negative groups, the pCR rate in the sensitive group was significantly higher than that in the non-sensitive group ($P < 0.001$, $P < 0.001$)（Supplementary Figure 4 C-D）. HER2-positive tumors are known to achieve pCR more readily, and among all the cases in the study (N = 1035), the pCR rate was 42.7% for HER2-positive tumors versus 12.7% for HER2-negative tumors. However, in HER2+ patients, the pCR rate was 42.7% in the sensitive group and 30.2% in the non-sensitive group, and the difference between the two groups was not statistically significant ($P = 0.051$). In the HER2 negative group, the pCR rate in the IPM-sensitive group was also significantly higher than that in the IPM-non-sensitive group (34.7% vs 7.2%, $P < 0.001$) (Supplementary Figure 4 E-F).

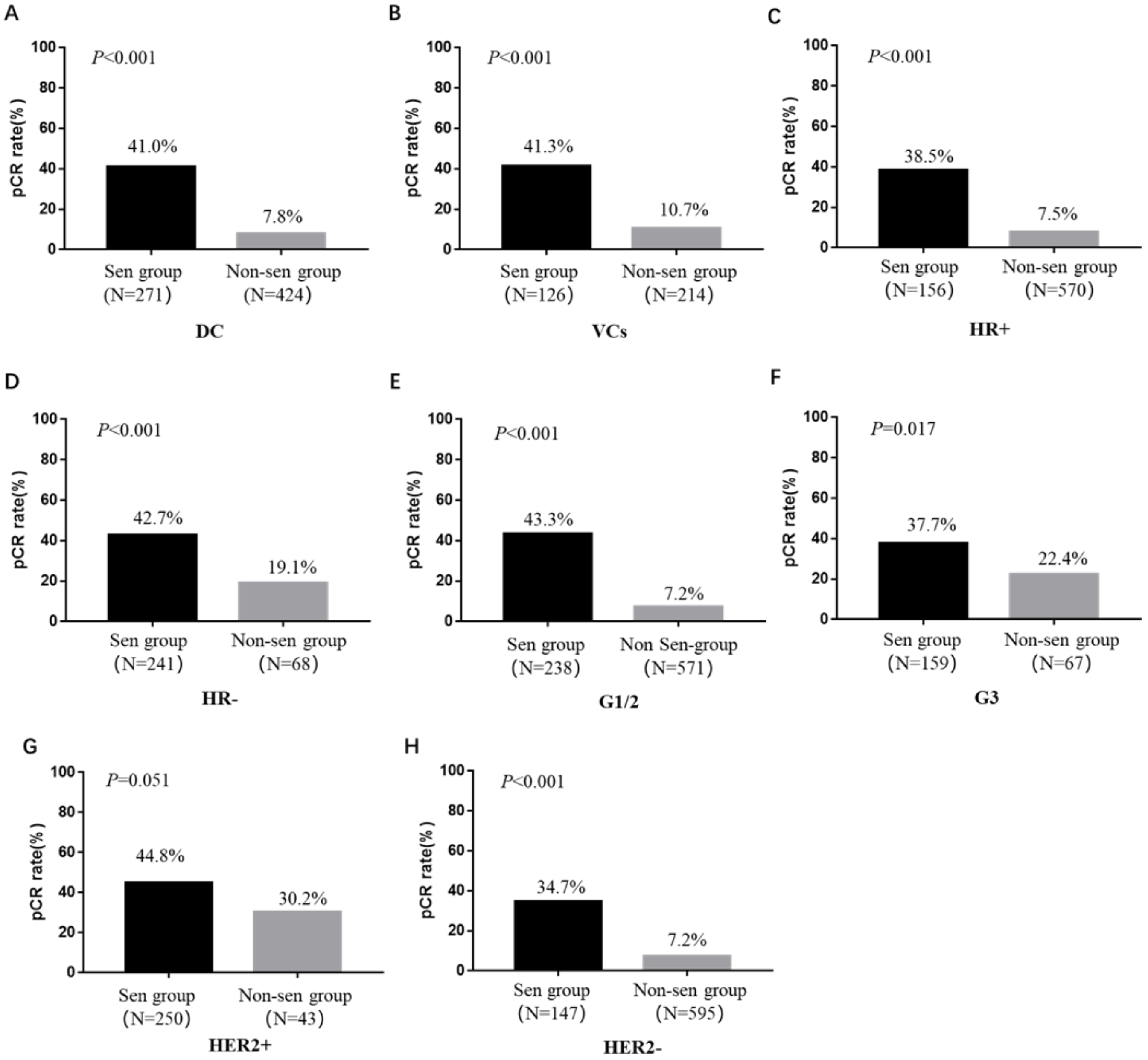

Supplementary Figure 4 The pCR rate in different breast cancer subgroups

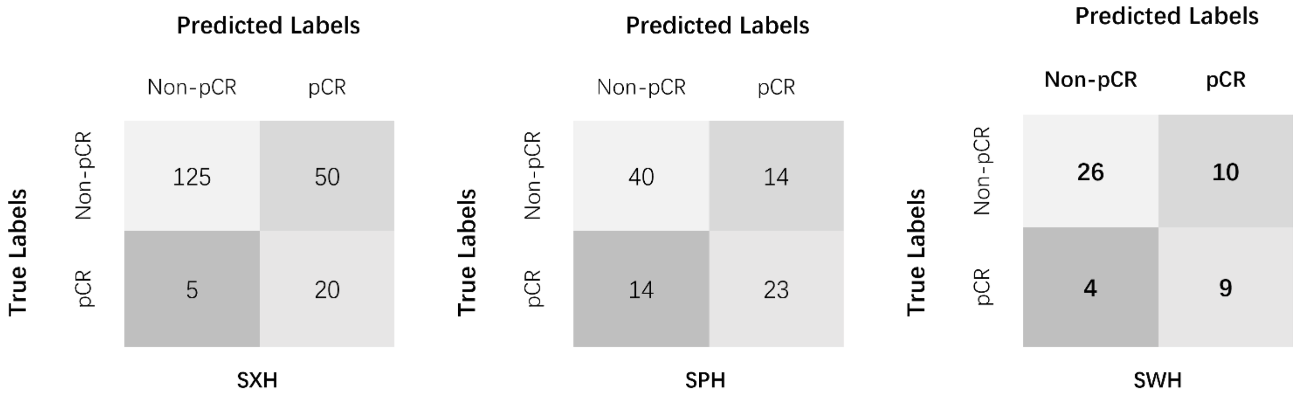

Supplementary Figure 5 Confusion matrix of IPM for predicting pCR in three external validation sets

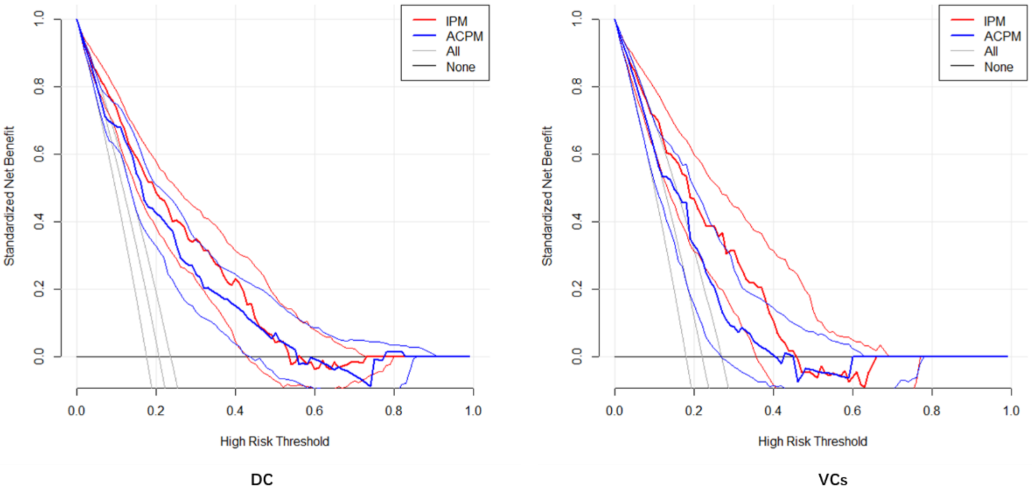

Supplementary Figure 6 Decision Curves of IPM and ACPM in the discovery cohort (DC) and the validation cohorts (VCs)

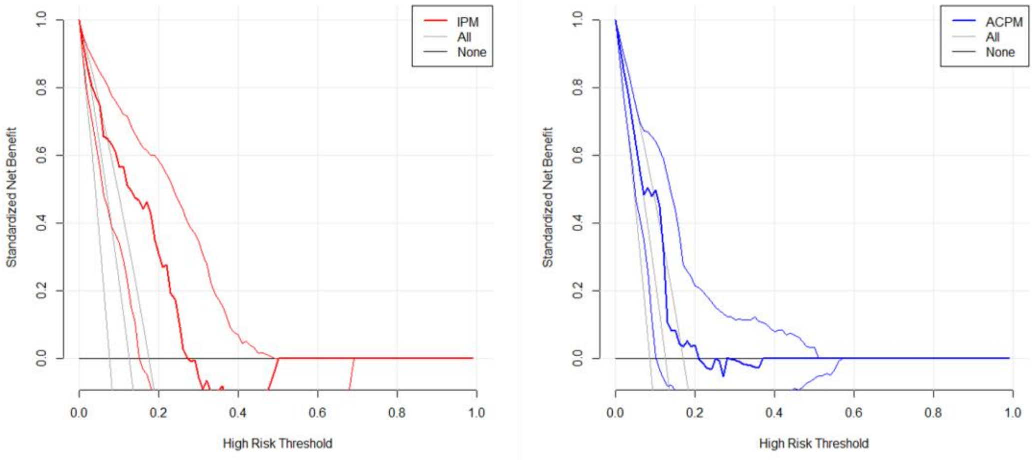

Supplementary Figure 7 Decision Curves of IPM and ACPM in V1 cohort (SXH)

Supplementary Note II

In addition, we separately compared the classification performance of the two models, IPM and ACPM, under the best threshold. Except for PPV, the other performance indexes of IPM were higher than 0.700. ACC, specificity and PPV were higher than ACPM, but the sensitivity was slightly lower. Both IPM and ACPM showed high NPV (0.922 and 0.934) (Supplementary Table 8). To facilitate the presentation of the results, three external data sets V1, V2, and V3 were integrated and calculated (Supplementary Table 9). Similarly, IPM presented higher ACC, specificity, PPV and AUC values than ACPM in VCs, but both models had higher NPV.

Supplementary Table 8 Comparison of ACPM and IPM in the discovery cohort

	IPM		ACPM	
	**Value**	**95% CI**	**Value**	**95% CI**
**ACC**	0.722	0.690-0.766	0.660	0.624-0.705
**Sensitivity**	0.774	0.700-0.855	0.833	0.773-0.894
**Specificity**	0.712	0.684-0.750	0.615	0.576-0.664
**PPV**	0.412	0.375-0.451	0.361	0.328-0.402
**NPV**	0.922	0.898-0.944	0.934	0.911-0.961
**AUC**	0.816	0.787-0.861	0.790	0.751-0.829

Supplementary Table 9 Comparison of ACPM and IPM in the validation cohorts

	IPM		ACPM	
	**Value**	**95% CI**	**Value**	**95% CI**
**ACC**	0.714	0.690-0.750	0.615	0.577-0.657
**Sensitivity**	0.698	0.636-0.768	0.813	0.750-0.877
**Specificity**	0.721	0.682-0.755	0.558	0.506-0.614
**PPV**	0.412	0.381-0.450	0.343	0.315-0.373
**NPV**	0.893	0.871-0.917	0.914	0.883-0.945
**AUC**	0.780	0.726-0.833	0.706	0.645-0.768

Supplementary Note III

The proportions of 22 immune cells in four data sets (GSE25066, GSE20194, GSE41998 and GSE20271) were estimated by CIBERSORT, and the distribution differences of immune cells in pCR and non-pCR groups in each data set were compared. In GSE25066 dataset, the percentage of T cells follicular helper (Tfh) ($P < 0.001$), M1 macrophages ($P < 0.01$), activated natural killer cells (NK) ($P < 0.01$) in the pCR group were significantly higher than those in the control group. NK) ($P < 0.05$) was significantly higher, while Treg, M2 macrophages and resting mast cells were significantly lower than those in non-pCR group ($P < 0.05$). The differences in the distribution of each subtype of immune cells between the pCR and non-pCR groups in the other three data sets are shown in Figure 6B-D. Comprehensive comparison found that only three kinds of immune cells had differences in the pCR and non-pCR groups in ≥ 3 datasets, including **Treg, M1 macrophages and resting mast cells** ($P < 0.05$). In GSE25066, GSE20194 and GSE20271, Treg cells in non-pCR group were higher than those in pCR group, while M1 macrophages in pCR group were significantly higher than those in non-pCR group. In GSE25066, GSE20194 and GSE41998, the resting mast cell infiltration density in non-pCR group was significantly higher than that in pCR group ($P < 0.05$).

Supplementary Table 10 The distributions of immune cells among pCR and non-pCR groups

**Immune cell**		**Patients (N=388)**		***P*-value**
		**pCR N=104 (%)**	**Non-pCR N=284 (%)**	
**Treg**	Absent	33（31.7%）	127（44.7%）	**0.027**
	Present	71（68.3%）	157（55.3%）	
**M1 macrophage**	Absent	55（52.9%）	153（53.9%）	0.909
	Present	49（47.1%）	131（46.1%）	

Immune cell		Patients (N=388)		*P*-value
		pCR N=104 (%)	Non-pCR N=284 (%)	
**M2 Macrophage**	N/mm^2	34（0~206）	32（0~181）	0.146
**Mast cell**	N/mm^2	14（1~80）	17（0~143）	**0.026**

In the pCR group, Treg was absent in 31.7% of the patients and was present in 68.3% of the patients, which was higher than that in the non-pCR group ($P = 0.027$). The expression of M1 macrophages in pCR and non-pCR groups was 47.1% and 46.1%, respectively, and there was no significant difference between the two groups ($P = 0.909$). Similarly, the median M2 macrophage infiltration density was 34 cells /mm^2 in pCR and 32 cells /mm^2 in non-pCR, with no significant difference ($P = 0.146$). The density of mast cells in the pCR group was 13 cells /mm^2 (range: 1 to 80), which was significantly lower than the distribution in the non-pCR group (median: 17 cells /mm^2, range: 0 to 143) ($P = 0.026$). Therefore, Treg and mast cells were significantly correlated with pCR, but neither M1 nor M2 macrophages were significantly correlated with pCR in this experiment (Figure 6E-H and Supplementary Table 10).

The correlations between the four immune cells and clinical stage, HR, HER2, Ki67, histological grade and sTILs were analyzed (Figure 6I). There was a significant correlation between HR status and mast cells (R = 0.23, $P < 0.001$), that is, the density of mast cells in HR positive tumors was higher than that in HR negative tumors. However, HR status was not significantly correlated with Treg and M1/M2 macrophages ($P = 0.155$, $P = 0.257$, $P = 0.132$). Ki67 was positively correlated with Treg and M2 macrophage infiltration (R = 0.150, $P = 0.004$; R = 0.150, $P = 0.003$). There was a slight negative correlation between Ki67 expression and mast cells, that is, breast cancer with a high expression of Ki67 may have a lower density of mast cells ($P = 0.011$). Compared with low grade tumors, Treg and M1/M2 macrophages were more infiltrated in high grade tumors ($P < 0.001$, $P = 0.025$, $P < 0.001$), but there was no correlation between grade and mast cells ($P = 0.971$). Treg and M2 macrophages were also positively correlated with sTILs density (R = 0.28, $P < 0.001$; R = 0.31, $P < 0.001$), but we did not find any correlation between mast cells and M1 macrophages and sTILs. Similarly, HER2 and clinical stage were not correlated with the abundance of the four immune cell infiltrations.

Supplementary Note IV

Checklist for Artificial Intelligence in Medical Imaging (CLAIM)			
Section/Topic	No.	Item	Yes/No
TITLE or ABSTRACT			
	1	Identification as a study of Al methodology, specifying the category of technology used (e.g., deep learning)	√
ABSTRACT			
	2	Structured summary of study design, methods, results, and conclusions	√
INTRODUCTION			
	3	Scientific and clinical background, including the intended use and clinical role of the Al approach	√
	4	Study objectives and hypotheses	√
METHODS			
Study Design	5	Prospective or retrospective study	√
	6	Study goals, such as model creation, exploratory study, feasibility study, noninferiority trial	√
Data	7	Data sources	√
	8	Eligibility criteria: how, where, and when potentially eligible participants or studies were identified (e.g. symptoms, results from previous tests, inclusion in registry, patient-care setting, location, dates)	√
	9	Data preprocessing steps	√
	10	Selection of data subsets, if applicable	√
	11	Definitions of data elements, with references to common data elements	√
	12	De-identification methods	√
	13	How missing data were handled	√
Ground Truth	14	Definition of ground truth reference standard, in sufficient detail to allow replication	√
	15	Rationale for choosing the reference standard (if alternatives exist)	√
	16	Source of ground truth annotations; qualifications and preparation of annotators	√
	17	Annotation tools	√
	18	Measurement of inter- and intrarater variability; methods to mitigate variability and/or resolve discrepancies	√
Data Partitions	19	Intended sample size and how it was determined	√
	20	How data were assigned to partitions; specify proportions	√
	21	Level at which partitions are disjoint (eg, image, study, patient, institution)	√
Model	22	Detailed description of model, including inputs, outputs, all intermediate layers and connections	√
	23	Software libraries, frameworks, and packages	√
	24	Initialization of model parameters (eg, randomization, transfer learning)	√

Training	25	Details of training approach, including data augmentation, hyperparameters, number of models trained	√
	26	Method of selecting the final model	√
	27	Ensembling techniques, if applicable	√
Evaluation	28	Metrics of model performance	√
	29	Statistical measures of significance and uncertainty (e.g., confidence intervals) IEE	√
	30	Robustness or sensitivity analysis	√
	31	Methods for explainability or interpretability (e.g., saliency maps) and how they were validated	√
	32	Validation or testing on external data	√
RESULTS			
Data	33	Flow of participants or cases, using a diagram to indicate inclusion and exclusion	√
	34	Demographic and clinical characteristics of cases in each partition	√
Model Performance	35	Performance metrics for optimal model(s) on all data partitions	√
	36	Estimates of diagnostic accuracy and their precision (such as 95% confidence intervals)	√
	37	Failure analysis of incorrectly classified cases	√
DISCUSSION			
	38	Study limitations, including potential bias, statistical uncertainty, and generalizability	√
	39	Implications for practice, including the intended use and/or clinical role	√
OTHER INFORMATION			
	40	Registration number and name of registry	×
	41	Where the full study protocol can be accessed	√
	42	Sources of funding and other support; role of funders	√

[1] AURELIO Y S, DE ALMEIDA G M, DE CASTRO C L, et al. Learning from imbalanced data sets with weighted cross-entropy function [J]. Neural processing letters, 2019, 50(2): 1937-49.

[2] THEODORIDIS S. Chapter 5 - Stochastic Gradient Descent: The LMS Algorithm and its Family [M]//THEODORIDIS S. Machine Learning. Oxford; Academic Press. 2015: 161-231.

[3] YANG Y, LV H, CHEN N, et al. Local Minima Found in the Subparameter Space Can Be Effective for Ensembles of Deep Convolutional Neural Networks [J]. Pattern Recognition, 2020, 109: 107582.

[4] YONGQUAN Y, HAIJUN L, NING C, et al. FTBME: feature transferring based multi-model ensemble [J]. Multimedia Tools and Applications, 2020, 79(25): 18767-99.

[5] YANG Y, LV H, CHEN N. A survey on ensemble learning under the era of deep learning [J]. Artificial Intelligence Review, 2022: 1-45.

[6] NEWMAN A M, LIU C L, GREEN M R, et al. Robust enumeration of cell subsets from tissue expression profiles

[J]. Nature methods, 2015, 12(5): 453-7.
[7] GENTLES A J, NEWMAN A M, LIU C L, et al. The prognostic landscape of genes and infiltrating immune cells across human cancers [J]. Nat Med, 2015, 21(8): 938-45.
[8] YUNNA C, MENGRU H, LEI W, et al. Macrophage M1/M2 polarization [J]. European journal of pharmacology, 2020, 877: 173090.
[9] ITALIANI P, BORASCHI D. From monocytes to M1/M2 macrophages: phenotypical vs. functional differentiation [J]. Frontiers in immunology, 2014, 5: 514.
[10] MARTINEZ F O, GORDON S. The M1 and M2 paradigm of macrophage activation: time for reassessment [J]. F1000prime reports, 2014, 6.
[11] LAOUI D, MOVAHEDI K, VAN OVERMEIRE E, et al. Tumor-associated macrophages in breast cancer: distinct subsets, distinct functions [J]. International Journal of Developmental Biology, 2011, 55(7-8-9): 861-7.
[12] SOUSA S, BRION R, LINTUNEN M, et al. Human breast cancer cells educate macrophages toward the M2 activation status [J]. Breast cancer research, 2015, 17(1): 1-14.
[13] CHEN Y, ZHANG S, WANG Q, et al. Tumor-recruited M2 macrophages promote gastric and breast cancer metastasis via M2 macrophage-secreted CHI3L1 protein [J]. Journal of hematology & oncology, 2017, 10(1): 1-13.
[14] MUKHTAR R A, NSEYO O, CAMPBELL M J, et al. Tumor-associated macrophages in breast cancer as potential biomarkers for new treatments and diagnostics [J]. Expert review of molecular diagnostics, 2011, 11(1): 91-100.
[15] VäYRYNEN J P, VORNANEN J O, SAJANTI S, et al. An improved image analysis method for cell counting lends credibility to the prognostic significance of T cells in colorectal cancer [J]. Virchows Archiv, 2012, 460(5): 455-65.
[16] ABRàMOFF M D, MAGALHãES P J, RAM S J. Image processing with ImageJ [J]. Biophotonics international, 2004, 11(7): 36-42.
[17] LI B, LI F, LIU Z, et al. Deep learning with biopsy whole slide images for pretreatment prediction of pathological complete response to neoadjuvant chemotherapy in breast cancer: A multicenter study [J]. Breast (Edinburgh, Scotland), 2022, 66: 183-90.
[18] LI F, YANG Y, WEI Y, et al. Predicting neoadjuvant chemotherapy benefit using deep learning from stromal histology in breast cancer [J]. NPJ breast cancer, 2022, 8(1): 124.
